# High-fat diet impairs the dendritic morphology of hippocampal CA1 pyramidal neurons in male but not female mice

**DOI:** 10.3389/fnut.2025.1687060

**Published:** 2025-11-17

**Authors:** Shu-Fang Teng, Ming Tatt Lee, Li-Jen Lee, Ling-Ling Hwang, Ching-Ping Chen, Hsin-Jung Lee, Chiung-Tong Chen, Lih-Chu Chiou

**Affiliations:** 1 Department and Graduate Institute of Pharmacology, National Taiwan University, Taipei, Taiwan; 2 Office of Postgraduate Studies, UCSI University, Kuala Lumpur, Malaysia; 3 College of Health Sciences, Chang-Jung Christian University, Tainan, Taiwan; 4 Graduate Institute of Brain and Mind Sciences, College of Medicine, National Taiwan University, Taipei, Taiwan; 5 Graduate Institute of Anatomy and Cell Biology, College of Medicine, National Taiwan University, Taipei, Taiwan; 6 Department of Physiology, College of Medicine, Taipei Medical University, Taipei, Taiwan; 7 Institute of Biotechnology and Pharmaceutical Research, National Health Research Institutes, Miaoli, Taiwan; 8 School of Pharmacy, College of Pharmacy, China Medical University, Taichung, Taiwan; 9 Graduate Institute of Acupuncture Science, China Medical University, Taichung, Taiwan

**Keywords:** high-fat diet, sex difference, dendritic spine density, dendritic arbor, hippocampal CA1 neurons

## Abstract

**Background:**

Obesity is associated with cognitive function impairment. We previously found that male, but not female, mice have poorer performance in learning and memory tasks and impaired hippocampal synaptic plasticity after long-term high-fat diet (HFD) consumption, compared to regular chow-fed counterparts. To elucidate the potential morphological mechanism(s), here we further performed morphometric analysis of hippocampal dendritic morphology and complexity in HFD and control groups of both sexes.

**Methods:**

C57BL/6 J mice with both sexes were fed HFD (45% kcal% fat) after weaning for 12 months. Age-matched control mice were fed regular chows (13.5 kcal% fat). Morphometric analysis of Golgi-stained dendrites in hippocampal slices was performed to compare the dendritic morphology and complexity of CA1 pyramidal neurons between HFD and control groups in male and female mice.

**Results:**

Compared with the control group, HFD-fed male mice showed lower dendritic spine density in both apical and basal dendrites, and lesser dendritic complexity in basal dendrites, which was indicated by fewer bifurcation nodes, terminal endings and dendritic segments, and shorter total dendritic length. However, in female mice, HFD did not affect dendritic spine density and induced subtle changes in dendritic complexity. Nevertheless, in control groups, male mice inherently had higher dendritic spine density and more dendritic complexity than females.

**Conclusion:**

The present study provides the structural evidence, including the reduction of dendritic complexity and spine density, for HFD-induced male-specific functional impairments in hippocampal synaptic plasticity and memory performance.

## Introduction

Hippocampus is an evolutionarily conserved brain region crucial for navigation and episodic memory in mammals (1, 2). Morphological changes in this region are often associated with memory-loss conditions such as dementia and Alzheimer’s disease (AD) (3, 4). Especially in AD patients, obesity is one of the risk factors that negatively impact the hippocampal-dependent memory (5, 6). A neuroimaging study has shown that overweight adults, compared to normal controls, have a higher degree of hippocampal atrophy (7), a condition highly associated with memory impairment (8). This association was, surprisingly, also observed in children with obesity (9). Although the manifestation of memory loss usually commences in older adults, numerous studies have revealed detrimental changes in earlier life. The impact of juvenile obesity-induced memory impairment is across the lifespan (10, 11) and highly correlated with daily diet (12–14).

High-fat diet (HFD)-induced obesity models in rodents can recapitulate large parts of the pathogenesis of human obesity (15), compared to the genetically-manipulated obesity model, like *ob-ob* mice. In HDF-induced obesity models, impairment of hippocampal-dependent memory has been reported in juvenile/adolescent (16), middle-aged (17), and aged (18) rodents. Especially, in our previous study, we found a sex difference in the negative impact of HFD on the learning and memory performance of mice (17). Mice fed HFD after weaning until 1-year-old (HFD-mice), beside exhibited significant obesity-related metabolic changes, such as elevation of plasma glucose, cholesterol, insulin, leptin and adiponectin, also had poorer performance in hippocampus-dependent learning and memory tasks and impaired hippocampal synaptic plasticity, long-term potentiation (LTP), in the male, but not female, group (17). Our findings were in-line with the recent report (19) that male mice are more vulnerable than female juvenile mice in HFD-induced reduction of hippocampus-dependent aversive memory. Such interplay between obesity and sex has also been reported in AD patients (20).

Morphological changes in hippocampal dendritic spines are a histological biomarker of synaptic plasticity (21), which is crucial in memory formation and consolidation (22). For example, hippocampal CA1 dendritic spine density is directly proportional to the memory capacity of mice (23) and rats (24). The dendritic spine number and length of hippocampal CA1 neurons were decreased in an AD mouse model (25). However, so far, there is no morphological analysis for the impact of long-term HFD on hippocampal dendritic morphology between sexes, while the available literature revolves around the hippocampal dendritic spine density affected by obesogenic diet in male rodents (26–28). Therefore, in this study, we investigated whether long-term HFD induced changes, with relation to sex differences, in the dendritic morphology of hippocampal CA1 pyramidal neurons using morphometric analyses.

## Materials and methods

All experimental protocols were conducted in adherence to the ethical guidelines approved by the Institutional Animal Care and Use Committees in College of Medicine, National Taiwan University and in National Health Research Institute, Taiwan.

### Animals

HFD-induced obese C57BL/6 J mice and normal diet-fed mice in both sexes were raised as reported previously (17). The mice were purchased from the National Laboratory Animal Center, Taiwan. Briefly, both male and female mice were randomly divided into HFD and normal diet groups. Mice in HFD and normal diet groups were fed rodent chows consisting of 45% (research diets Inc., NJ, United States, product ID: D12451) and 13.5% (LabDiet^®^, PA, United States. product ID: 5010) kcal% fat, respectively, after weaning (3–4 weeks old), for 12 months. The body weight was measured individually twice and once a week, respectively, before and after 12 weeks of age. Mice were housed in groups of 5 per cage under controlled temperature and 12:12 h light–dark cycle with abovementioned diets and water *ad libidum*

### Golgi-Cox impregnation

After deeply anesthetized with 5% isoflurane, mice were sacrificed by intracardiac perfusion with 4% paraformaldehyde. The brains were isolated and post-fixed for later morphological studies. Brain sections of 200 μm-thick containing the hippocampus were sliced horizontally with Microslicer (DTK-1000, D. S. K., Japan) and collected.

The Golgi-Cox impregnation method was used for visualizing dendritic structures as reported previously (29) with modifications. Briefly, hippocampal slices were immersed into an impregnation solution, which was prepared by mixing solution A (1.0 g potassium dichromate and 1.0 g mercuric chloride in 85 mL water) and solution B (0.8 g potassium chromate and 0.5 g sodium tungstate in 20 mL water), at room temperature for 2 weeks. Sections were then collected and reacted with 15% ammonium hydroxide for 2 min and rinsed thoroughly in distilled water. Subsequently, sections were placed in diluted rapid fixer solution (1:5; Ilford, Marly, Switzerland) for 10 min and rinsed thoroughly in distilled water. Finally, all sections were mounted with a glycerol-based mounting medium.

### Morphometric analysis

#### Dendritic spines

The dendritic spines from both basal and apical dendrites of Golgi-Cox-impregnated hippocampal CA1 pyramidal neurons were captured with an Olympus light microscope (Olympus BX51, Tokyo, Japan) under a 60 × lens, and their numbers were counted using the ImageJ software (NIH, MA, United States). For basal dendrites, we counted spine numbers in the dendritic segments of orders over three. Since the spine density is usually low and varied in the primary and secondary orders, we chose segments over order 3 to reduce the intra-group variation (30). For apical dendrites, spine numbers beyond the distance of 150–200 μm apart from the soma were counted, as the spine density in the proximal segments (beyond 50 μm from the soma) is usually low and varied (31).

#### Dendrites

The morphology of Golgi-Cox-impregnated pyramidal neurons in the CA1 region was examined under a 20 × lens of the Olympus BX51 light microscope. The image stacks were captured with 1.5 μm Z-axis interval under the control of the Stereo Investigator system (MicroBrightField, MBF Bioscience, VT, United States). The morphology of CA1 pyramidal neurons with basal and apical dendrites was reconstructed by the Neurolucida software (MBF Bioscience). Morphometric analyses of basal dendrites of CA1 pyramidal neurons were performed according to the anatomical definition (32). The dendrites derived directly from the soma are the first-order branches or primary dendrites. The daughter branches arising from that are second-order branches, and so on. The highest branching order was determined by the maximum order of the selected neurons. The point at which a dendrite gives rise to two daughter branches was defined as a bifurcation node. The termination of a dendrite was counted as a terminal ending. The portion between two bifurcation nodes was defined as a segment. The Neurolucida Explorer program was used to count the numbers of primary dendrites, bifurcation nodes, terminal endings, and the highest orders of dendritic branching of the collected CA1 neurons in various groups. Due to the steric distribution of lengthy apical dendrites, it was technically challenging to obtain sufficient CA1 pyramidal neurons with complete apical dendrites. We only performed morphometric analysis for basal dendrites.

### Statistical analysis

**Figure 1:**
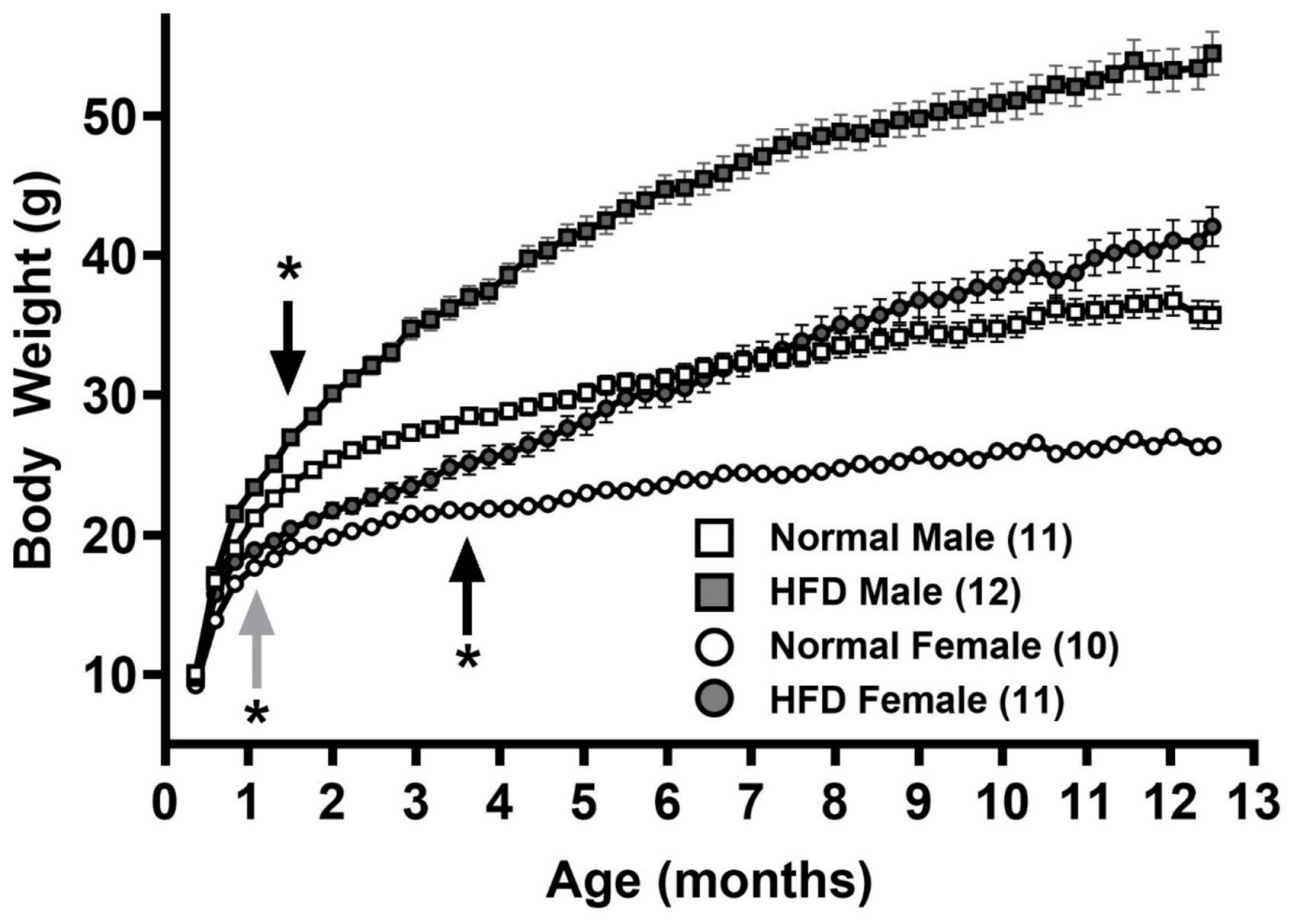
Growth curves of male and female mice fed normal chow and high-fat diet (HFD), respectively. Body weights of male (squares) and female (circles) mice fed normal chow (empty symbols) or HFD (filled symbols) after weaning (P21) for 1 year were measured twice and once a week, respectively, before and after 12 weeks of age. Data are mean ± SEM *p < 0.05 vs. the sex-specific control group (black arrows) or vs. the control male group (gray arrow) from the indicated ages (Two-way ANOVA followed by Bonferroni’s *post-hoc* test.) The numbers in parentheses are the numbers of animals in each group.

**Figure 2:**
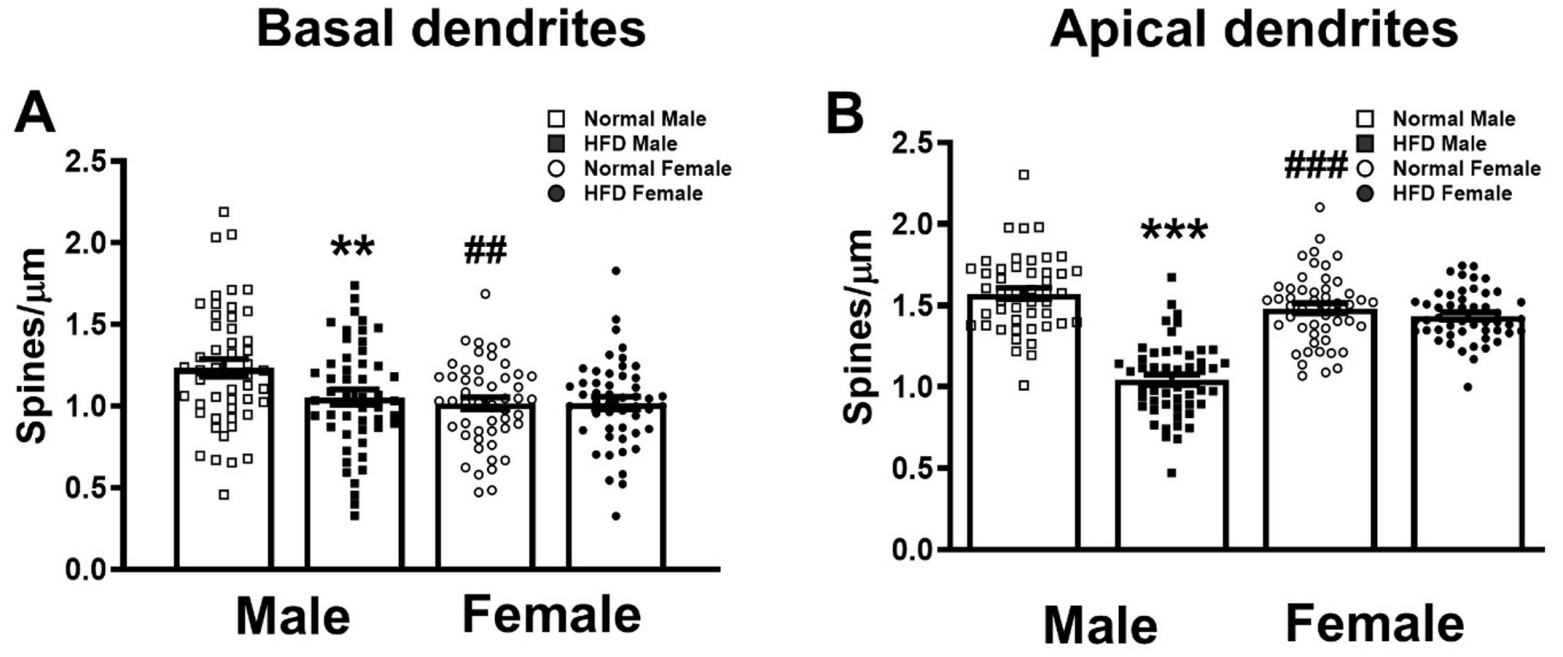
Effects of HFD on the spine density of basal and apical dendrites of hippocampal CA1 neurons. The dendritic spines of basal **(A)** and apical **(B)** dendrites of CA1 pyramidal neurons in both sexes of HFD-fed mice (HFD♂ and HFD♀) and normal control mice (NC♂ and NC♀). Dendritic spines from dendritic segments collected from each group were analyzed by Image J. Data are the mean ± SEM. n: the number of dendritic segments. **p < 0.01, ***p < 0.001 vs. the sex-specific control group, ^##^*p* < 0.01, ^###^*p* < 0.001 vs. the control male group, two-way ANOVA followed by Bonferroni’s *post-hoc* test.

**Figure 3:**
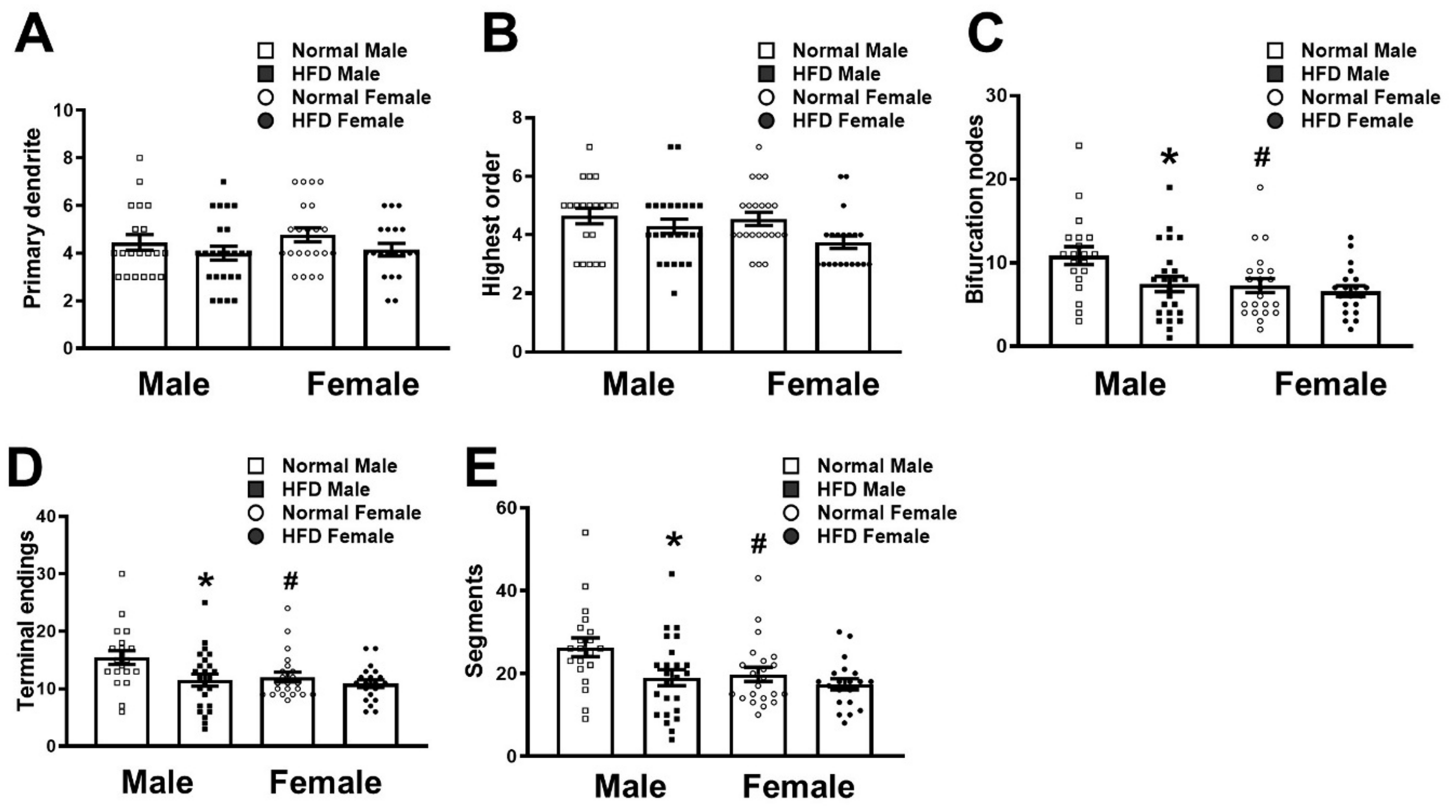
Morphometric analysis of the branching pattern of basal dendrites of CA1 pyramidal neurons in different groups. The number of primary dendrites **(A)**, the highest dendritic branching order **(B)**, the number of bifurcation nodes, terminal endings **(D)** and dendritic segments **(E)** in the basal dendrites of CA1 pyramidal neurons collected from HFD-fed mice (HFD♂, *n* = 24 neurons from 3 animals; HFD♀, *n* = 20 neurons from 6 animals) and normal control mice (NC♂, *n* = 20 neurons from 3 animals; NC♀, *n* = 22 neurons from 5 animals) were analyzed by the Neurolucida Explorer software. Data are the mean ± SEM. *p < 0.05 vs. the sex-specific control group. ^#^*p* < 0.05 vs. the control male group, two-way ANOVA followed by Bonferroni’s *post-hoc* test.

Data are expressed as mean ± SEM. For body weight comparisons (Figure 1), we employed two-way ANOVA with repeated measures over time followed by Bonferroni’s *post-hoc* test. For dendritic spine density (Figure 2), the data was analyzed with two-way ANOVA over diet and sex factors, followed by Bonferroni’s *post-hoc* test, and passed the Shapiro–Wilk normality test. For morphological analyses of dendrites (Figure 3), the data underwent logarithmic transformations then analyzed with two-way ANOVA over diet and sex factors, followed by Bonferroni’s *post-hoc* test, and passed the Shapiro–Wilk normality test. Spearman’s test was used to test for heteroscedasticity in these datasets. *p* < 0.05 was considered to be significant.

## Results

### Body weight

Figure 1 shows the growth curves of normal diet control-male (empty squares), normal diet control-female (empty circles), HFD-male (filled squares) and HFD-female (filled circles) groups of mice. A two-way ANOVA with repeated measures over Age shows main effects of Age [*F*(52, 2080) = 841.5, *p* < 0.001, η^2^*p* = 0.955], Group [*F*(3, 40) = 115.0, *p* < 0.001, η^2^*p* = 0.958] and a significant interaction between Age and Group [*F*(156, 2080) = 47.90, *p* < 0.001, η^2^*p* = 0.782]. *Post hoc* analyses with the Bonferroni’s test show that in normal diet control groups, male mice grew significantly heavier than females starting at 32-day old (upward gray arrow, Figure 1, *p* = 0.032), and throughout the feeding duration till 1-year-old (*p* < 0.001, main Group effect). On the other hand, consistent with our previous study (17), HFD groups gained significantly more weight than control groups in both sexes. Compared with control groups of the same sex, HFD-male mice significantly gained more weights starting at 43-day old (downward black arrow, Figure 1, *p* = 0.041), in contrast with HFD-females at 109-day old (upward black arrow, Figure 1, *p* = 0.042).

### Effects of HFD on the spine density of hippocampal CA1 apical and basal dendrites

**Figure 4:**
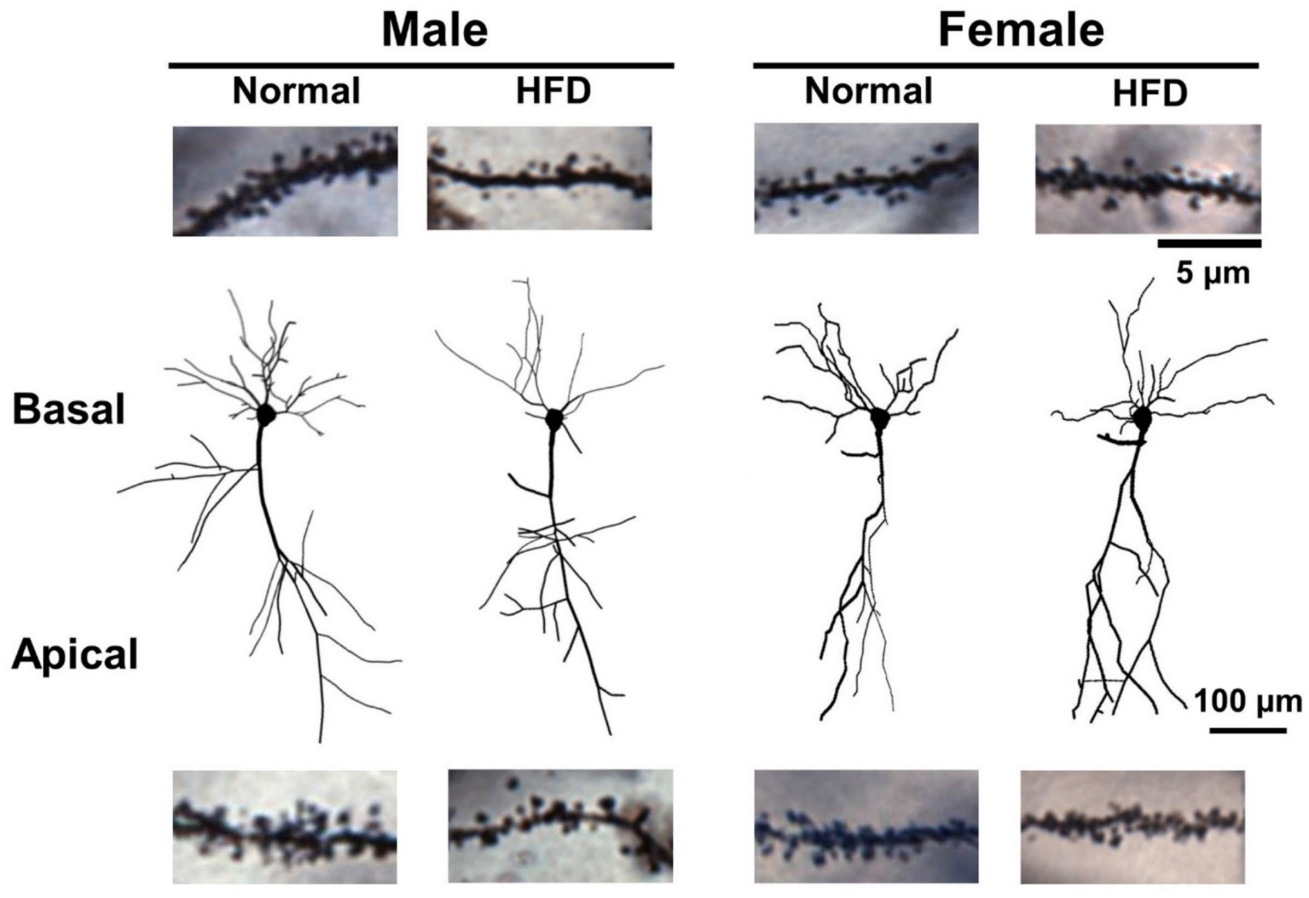
The dendritic morphology and dendritic spines of hippocampal CA1 pyramidal neurons of control males, control females, HFD-males and HFD-females. The Neurolucida-reconstructed morphology of Golgi-Cox impregnated CA1 pyramidal neurons with well impregnated dendrites collected from four groups of mice. The dendritic spines were omitted in this illustration. Representative photographs of basal (upper) and apical (lower) dendritic segments containing dendritic spines were collected from CA1 neurons of different groups. Scale bar: 100 μm for CA1 neurons and 5 μm for dendritic segments.

Figure 4 shows the morphology of Neurolucida-reconstructed CA1 pyramidal neurons with well-impregnated apical and basal dendrites in hippocampal sections taken from control-male, HFD-male, control-female and HFD-female groups of mice, respectively. Representative micrograms of dendritic spines on basal dendrites (upper panel) and apical dendrites (lower panel) from four groups were also shown.

First, we compared the dendritic spine density of basal and apical dendrites among four groups. The detailed statistical parameters of datasets in Figures 2, 3 are listed in Supplementary Table S1. Two-way ANOVA followed by Bonferroni’s *post-hoc* test shows that, in basal dendrites (Figure 4 upper panel), HFD-males had significantly lower dendritic spine density than control-males (open square bar vs. filled square bar, Figure 2A, *P* = 0.009, Bonferroni’s test). However, there was no significant difference in the basal spine density between HFD-female and control-female groups (Figure 4 upper panel; filled circle bar vs. open circle bar, Figure 2A, *P* = 0.999). Interestingly, in normal-diet control groups, males had significantly higher basal spine density than females control group (Figure 4 upper panel; open square bar vs. open circle bar, Figure 2A, *P* = 0.002, Bonferroni’s test). Similar trend was also observed in the apical dendrites (Figure 4 lower panel; Figure 2B). HFD significantly decreased the apical dendritic spine density in male mice (*p* < 0.001), but not in female mice (*p* > 0.999), as compared with the control group with the same-sex (Figure 2B, two-way ANOVA followed by Bonferroni’s *post-hoc* test). Interestingly, compared with females, control males also had a significantly higher spine density on apical dendrites (Figure 4 lower panel; Figure 2B open square bar vs. open circle bar, *p* < 0.001, Bonferroni’s test), as observed on basal dendrites.

### Effects of HFD on the morphology of basal dendrites of hippocampal CA1 neurons

Next, we compared the complexity of basal dendrites of hippocampal CA1 neurons in four groups of mice. Morphological analyses on the dendritic branching pattern using the Neurolucida Explorer software (Figure 3) showed that the HFD-male group (filled square bars) had significantly fewer bifurcation nodes (Figure 3C, *P* = 0.017), terminal endings (Figure 3D, *P* = 0.035) and dendritic segments (Figure 3E, *P* = 0.034, two-way ANOVA with Bonferroni’s *post-hoc* test) than the control male group (open square bars). Nevertheless, both groups had similar numbers of primary dendrites (Figure 3A, *P* > 0.999) and the highest branching order (Figure 3B, *P* = 0.583, Bonferonni’s *post-hoc* test). On the other hand, HFD did not induce a significant change in the branching pattern of basal dendrites in female mice (open circle bars vs. filled circle bars, Figure 3). Interestingly, as compared with the control male group, the basal dendrites of CA1 neurons in control females had fewer bifurcation nodes (Figure 3C, *P* = 0.018), terminal endings (Figure 3D, *P* = 0.05) and dendritic segments (Figure 3E, *P* = 0.045, Bonferroni’s *post-hoc* test).

These results suggest that middle age male mice have more dendritic arbors of CA1 pyramidal neurons than age-matched females, and that chronic HFD reduces the complexity of basal dendritic of CA1 pyramidal neurons preferentially in male mice, but not in females.

## Discussion

In continuation with our previous study that revealed a sex difference in the role of chronic HFD in memory impairment of middle-aged mice (17), the present study further disclosed its possible underlying morphological evidence in hippocampal CA1 pyramidal neurons. The complexity of dendritic arbors determines the number and distribution of receptive synaptic contacts (33, 34). In hippocampal CA1 pyramidal neurons, dendritic spines are the primary postsynaptic sites for excitatory neurotransmission (35, 36). The spine number and morphology of CA1 dendritic spines are vulnerable to the experience of the subject (35, 37, 38), and their changes are associated with memory encoding and retrieval (39, 40). Reduced dendritic spines and arbors in the hippocampal neurons have been reported in animals with memory impairment (34, 36, 41) and in patients with mild cognitive impairment and AD (34, 41–43). Therefore, in the present study, the dendritic spine density and dendritic arbors of hippocampal CA1 pyramidal neurons were chosen as the structural base for the functional implications of HFD. As summarized in Table 1, there was inherent sex difference in dendritic structures of CA1 pyramidal neurons in 1 year-old normal chow-fed mice. More importantly, our results demonstrated, for the first time, that 1 year of HFD feeding in male, but not female, mice after weaning leads to impairments of dendritic spine density and complexity in hippocampal CA1 pyramidal neurons.

**Table 1:** Comparisons of hippocampal CA1 dendritic morphology, synaptic plasticity and functions between different sexes and diets.

Comparisons	NC♂ vs. NC♀	HFD**♂** vs. NC♂	HFD**♀** vs. NC♀
Dendritic spine density			
Basal dendrites (Figure 2A)	NC♂ > NC♀	HFD**♂** *<* NC♂	HFD**♀** = NC♀
Apical dendrites (Figure 2B)	NC♂ > NC♀	HFD**♂** *<* NC♂	HFD**♀** = NC♀
Basal dendrite morphology			
Branching pattern (Figure 3)	NC♂ > NC♀	HFD**♂** < NC♂	HFD**♀** = NC♀
CA1-associated functions^a^			
Synaptic plasticity	NC♂ > NC♀	HFD**♂** *<* NC♂	HFD**♀** = NC♀
Memory performance	NC♂ > NC♀	HFD**♂** *<* NC♂	HFD**♀** = NC♀

NC♂, normal diet control male; NC♀, normal diet control female; HFD**♂**, high-fat diet male; HFD**♀**, high-fat diet female. Mice in the HFD group were fed HFD after weaning for 1 year. ^a^Based on the data in our previous study (17).

### Inherent sex difference in the dendritic spine density and morphology of hippocampal CA1 neurons of middle-aged mice

As summarized in Table 1, there was sex difference in the number of dendritic spines in both basal and apical dendrites as well as the arborization of basal dendrites of hippocampal CA1 neurons. Compared with female mice in the control group, males had a higher spine density in both basal and apical dendrites of hippocampal CA1 neurons (Figure 2). Besides, morphometric analysis also showed that the control male group had higher dendritic complexity in basal dendrites of hippocampal CA1 neurons (Figure 3). Taken together with our previous findings that control male mice, at the same age as employed here, exhibited better hippocampus-dependent memory and synaptic plasticity than age-matched control females (17), it can be speculated that higher inherent CA1 dendritic spine density in both basal and apical dendrites, and probably higher basal CA1 dendritic complexity may contribute to the inherent sex difference in hippocampus-dependent memory and synaptic plasticity in normal middle-aged mice. This observation is in-line with previous literature regarding the presence of sex difference in hippocampal-dependent memory processing in rodents (27, 44–47).

The inherent sex difference in the dendritic spine density and morphology of hippocampal CA1 neurons could largely be attributed to complex interactions of sex hormones, particularly estrogen and testosterone, which both promote the hippocampal dendritic spine density in the respective sex (48, 49). The plasma testosterone levels of male C57BL/6 mice did not drop drastically between ages 4 and 12 months (50). However, the plasma estrogen level decreased drastically in a similar age comparison (80), probably due to the gradual regression of ovarian function in female mice at 12 months.

### HFD reduces the dendritic spine density and arborization of hippocampal CA1 neurons in male, but not female, mice

The impact of HFD on the dendritic spine density of hippocampal CA1 pyramidal neurons was similar between basal and apical dendrites (Figure 2). Interestingly, these changes were only observed in male but not female mice (Table 1). Similarly, HFD-induced reduction of dendritic complexity was also observed in male but not in female mice (Table 1). These male-specific changes in hippocampal dendritic morphology after HFD are coincident with our previous finding in HFD-induced decline in the performance of hippocampal CA1-dependent memory tasks and hippocampal synaptic plasticity (17). It is therefore suggested that the loss of dendritic spines in both apical and basal dendrites and the impaired basal dendritic complexity of hippocampal CA1 neurons contribute to the male-specific change in memory performance affected by HFD.

In the present study, we could not obtain sufficient CA1 neurons with thoroughly impregnated apical dendrites for analysis due to the limitation that apical dendrites were mostly truncated during sample preparation. Nonetheless, several studies have reported that the morphometric complexity of both apical and basal dendrites of hippocampal CA1 neurons was simultaneously impacted under various conditions, such as chronic stress (51), radiation (52), and chemotherapy (53). It remains to be further elucidated whether long-term HFD may affect the morphometric complexity of CA1 apical dendrites in a similar trend to basal dendrites.

### HFD induces male-specific changes in hippocampal CA1 dendritic morphology, synaptic plasticity and memory performance

It has been reported that the hippocampus-dependent associative and spatial learning is associated with an increased dendritic spine density specifically in the basal, but not apical, dendrites of CA1 neurons (39, 40). Basal dendrites of CA1 neurons receive fewer inhibitory inputs from GABA interneurons than apical dendrites (54). and thus exhibit a lower threshold to induce LTP (55). Thus, basal dendrites may have a higher capacity for synaptic plasticity, the essence of memory formation (56). Nonetheless, apical dendritic spines of CA1 pyramidal cells are also crucial in memory consolidation and retrieval via LTP formation (57).

Here, we found that the spine density of both basal and apical dendrites of hippocampal CA1 neurons was decreased by chronic HFD for 12 months, which also impaired CA1 LTP and hippocampal-dependent memory performance, specifically in male mice (17). Thus, both basal and apical dendritic spine loss in male mice may contribute to their inferior performance in hippocampus-dependent memory tasks. Interestingly, previous studies showed that HFD administration in mice after weaning until 8–12 week-old enhanced LTP (58) and dendritic spine turnover (59) at hippocampal CA1 synapses, while disrupted LTP in younger (6–7 month-old) (60) and older (12 month-old) (17) mice. It would be interesting to study the gradual time-progression effect of HFD from 1 to 12 months of administration or longer on hippocampal morphology and electrophysiological properties in mice of both sexes in the future.

### Possible factors contributing to HFD-induced CA1 dendritic morphological changes in different sexes

Female gonadal hormones play an important role in modulating the plasticity of dendritic spines in the central nervous system (49), particularly in the hippocampus (61, 62). Li et al. (63) has shown that hippocampal-dependent memory and dendritic spines were reduced in ovariectomized female mice in a manner improved by estrogen. Luine and Frankfurt (64) also found reduced dendritic spines on both CA1 apical and basal dendrites of ovariectomized female rats, while estrogen restored the spines only on basal, but not on apical, dendrites. Thus, it can be postulated that the surge of gonadal hormones during the estrus cycle may provide neuroprotection against HFD-induced morphological impacts in female mice. However, in female mice, there was a fluctuation in hippocampal dendritic spine density between diestrus and proestrus phases (65). In the present study, the estrus staging of the tested female mice was not measured. Given that female mice are predominantly acyclic at the age of 13 months (66–68), the impact of the estrus cycle and estrogen level is expected to be minimal. Nonetheless, the influence of the estrus cycle in these cohorts of female mice still cannot be ruled out and thus poses a limitation of the current study, which requires further studies in the future.

Neuro-inflammation due to diet-induced obesity can be induced in several brain regions, including the hippocampus, and thus results in cognitive deficit in mice (26, 69). This is due to obesity-induced systemic inflammation that leads to increased infiltration of peripheral immune cells through the blood–brain barrier, elevated pro-inflammatory cytokines, and activation of microglia in the hippocampus (70). Microglial activation induced by dietary obesity can lead to reduced hippocampal dendritic spine density, due to “synaptic stripping” (26), i.e., an internalization of synaptic terminals induced by microglia (71). It was reported that the dendrites of CA1 neurons in male obese mice fed by HFD for 3 months suffered from the same fate, although females were not examined (72). However, 13-week-old male mice have been reported to inherently have more and larger microglia cells in the hippocampus than age-matched female mice (73). Besides, the number of microglia in the mouse hippocampus does not change with age (74). It remains to be further elucidated whether the inherently more hippocampal microglial cells in male mice may result in more microglia activation and synaptic stripping after neuroinflammation due to chronic HFD-induced obesity.

Moreover, current trends indicate that the microbiota-gut-brain axis may influence cognitive functions. Long-term consumption of a high-fat diet, recognized for its impact on cognitive function (17, 75), can also alter microbiota composition (76). However, a previous preclinical study indicates that, irrespective of microbiota composition, a high-fat diet will inevitably lead to obesity (77). Nevertheless, it remained unclear if the cognitive functions, particularly those reliant on the hippocampus, are influenced by the composition of the microbiota. HFD has been demonstrated to affect the microbiome in a sex-dependent manner (78), similar to HFD-induced obesity (17, 75). Although many studies indicate estrogen-dependent variations in HFD-altered gut microbiota (78, 79), no research has directly compared the microbiota compositions of males and females in relation to hippocampus-dependent cognitive function. This may necessitate additional research on the potential sexual dimorphism in the microbiome alterations related to HFD-induced obesity and cognitive impairment.

## Conclusion

The present study provides the morphological evidence showing the sex difference in CA1 neurons that could be linked to the impairment of memory performance in chronic HFD-induced obese mice. It is suggested that the deficits in the synaptic plasticity and learning and memory performance in obese male, but not female, mice are associated with the morphological changes, including the reductions in dendritic arbors and spine density, of hippocampal CA1 pyramidal neurons.

## Data Availability

The raw data supporting the conclusions of this article will be made available by the corresponding author based on reasonable request.
