# Supplementary material for: High-fat diet impairs the dendritic morphology of hippocampal CA1 pyramidal neurons in male but not female mice
Source: Front Nutr. 2025 Nov 17;12:1687060. doi: 10.3389/fnut.2025.1687060 (PMC12665603; doi:10.3389/fnut.2025.1687060)
Supplement: Supplementary file 1 [file Table_1.docx]

Table S1. Statistical parameters derived from two-way ANOVA in Figures 3 and 4.

| **Figure** | **Sex**  **(Main effect)** | **Diet**  **(Main effect)** | **Sex＊Diet** | **Shapiro-Wilk Test** | **Spearman’s Test** |
| --- | --- | --- | --- | --- | --- |
| **Fig. 3A**  Normal male  HFD male  Normal female  HFD female | *F*(1,196)=8.238; *P*=0.005; η^2^p = 0.04 | *F*(1,196)=4.057; *P*=0.045; η^2^p = 0.02 | *F*(1,196)=4.276; *P*=0.04; η^2^p = 0.021 | *W*=0.994; *P*=0.596 | *r_s_*(196)=0.098, *P*=0.085 |
| **Fig. 3B**  Normal male  HFD male  Normal female  HFD female | *F*(1,193)=24.24; *P*<0.001; η^2^p = 0.112 | *F*(1,193)=62.32; *P*<0.001; η^2^p = 0.244 | *F*(1,193)=88.87; *P*<0.001; η^2^p = 0.315 | *W*=0.990; *P*=0.210 | *r_s_*(193)=0.021, *P*=0.389 |
| **Fig. 4A**  Normal male  HFD male  Normal female  HFD female | *F*(1,76)=0.952; *P*=0.332; η^2^p = 0.012 | *F*(1,76)=3.24; *P*=0.076; η^2^p = 0.041 | *F*(1,76)=0.002; *P*=0.965; η^2^p = 2.61 x 10^-5^ | *W*=0.974; *P*=0.099 | *r_s_*(76)=0.076, *P*=0.253 |
| **Fig. 4B**  Normal male  HFD male  Normal female  HFD female | *F*(1,81)=1.631; *P*=0.205; η^2^p = 0.020 | *F*(1,81)=6.351; *P*=0.014; η^2^p = 0.073 | *F*(1,81)=1.025; *P*=0.314; η^2^p = 0.012 | *W*=0.973; *P*=0.066 | *r_s_*(81)=0.075, *P*=0.248 |
| **Fig. 4C**  Normal male  HFD male  Normal female  HFD female | *F*(1,80)=3.331; *P*=0.071; η^2^p = 0.040 | *F*(1,80)=4.701; *P*=0.033; η^2^p = 0.055 | *F*(1,80)=2.736; *P*=0.102; η^2^p = 0.033 | *W*=0.946; *P*=0.094 | *r_s_*(80)=0.109, *P*=0.161 |
| **Fig. 4D**  Normal male  HFD male  Normal female  HFD female | *F*(1,80)=1.253; *P*=0.266; η^2^p = 0.015 | *F*(1,80)=6.289; *P*=0.014; η^2^p = 0.073 | *F*(1,80)=2.638; *P*=0.108; η^2^p = 0.032 | *W*=0.977; *P*=0.140 | *r_s_*(80)=0.087, *P*=0.217 |
| **Fig. 4E**  Normal male  HFD male  Normal female  HFD female | *F*(1,78)=1.553;  *P*=0.216; η^2^p = 0.046 | *F*(1,78)=5.881; *P*=0.018; η^2^p = 0.067 | F(1,78)=2.343; *P*=0.130; η^2^p = 0.025 | *W*=0.980; *P*=0.225 | *r_s_*(78)=0.083, *P*=0.230 |
